# Strengthening the rural medical workforce: the need for a paradigm shift in protecting and growing the critical role of GP supervisors

**DOI:** 10.3389/fmed.2026.1868204

**Published:** 2026-06-29

**Authors:** Tamekha Develyn, Lachlan Van Schaik, Will Harvey, Zahra Ali, Julian Wright

**Affiliations:** Department of Rural Health, The University of Melbourne, Shepparton, VIC, Australia

**Keywords:** apprenticeship model of training, general practice supervision, general practitioner training, medical education, rural health workforce, rural medical workforce, workforce sustainability

## Abstract

Australia faces an entrenched and worsening maldistribution of general practitioners, leaving rural and remote communities with chronic workforce shortages and unacceptable barriers to accessing primary care in a timely manner. The apprenticeship-style model that underpins general practice training relies fundamentally on supervisors to provide clinical oversight, teaching, assessment and mentorship. This supervisory capacity, central to shaping registrar competence, professional identity and long-term rural practice is now one of the key rate-limiting factors in rural workforce development. That capacity is steadily eroding. An ageing workforce, post-pandemic attrition and escalating clinical demand are shrinking the pool of experienced supervisors, while growing dependence on international medical graduates and locums (less likely to supervise) further weakens the supervisory pipeline. Compounding this are persistent system-level failures: variable supervision standards, the late introduction of formal supervisor training, limited financial and structural incentives, and limited professional recognition. Together, these pressures constrain the ability of rural practices to train registrars at the scale required to address workforce shortages. Strengthening supervision is therefore not an adjunct consideration but a strategic necessity. Building a resilient supervisory workforce will require coordinated reform that embeds protected teaching time into routine practice, recognizes and remunerates supervisory work appropriately, expands dedicated educator roles, develops tiered and flexible supervision models and provides targeted support for women and international medical graduate clinicians. Without decisive investment in supervision, efforts to expand rural training pathways will exceed the system’s capacity to sustain them.

## Background

Australia faces an entrenched and escalating maldistribution of its general practitioner (GP) workforce. Although approximately 29% of Australians live in rural and remote regions, access to health services in these areas is consistently limited (1). The Modified Monash Model (MMM) classifies rurality from MMM 2 - 7, representing communities with lower population density, greater distance from services, and higher healthcare vulnerability (2). In these settings, GPs are not only the first point of healthcare access but are frequently the only local medical specialists, providing a broad scope of inpatient, primary, emergency and procedural care (3, 4).

In Australia, general practitioners are recognized medical specialists. Following completion of medical school, graduates undertake at least 1 year of supervised internship followed by additional prevocational hospital training, typically entering general practice vocational training during Postgraduate Year (PGY) 2–3. Registrars then complete three to 4 years of supervised community-based training and formal assessment within accredited practices. Fellowship pathways are overseen by the Royal Australian College of General Practitioners (RACGP) and the Australian College of Rural and Remote Medicine (ACRRM), leading to Fellowship of the Royal Australian College of General Practitioners (FRACGP) or Fellowship of the Australian College of Rural and Remote Medicine (FACRRM), respectively. While both pathways confer specialist recognition as a general practitioner, the FACRRM pathway incorporates a broader rural generalist scope of practice. Together, these colleges oversee accreditation standards, assessment, and certification for independent practice. Training is delivered predominantly through an apprenticeship-style model under the Australian General Practice Training (AGPT) program, whereby learning, assessment, and professional development are embedded within real-world general practice environments. Crucially, this model relies on the availability and capability of skilled GP supervisors to provide continuous clinical oversight, structured feedback, and mentorship. Without sufficient supervisory capacity, rural training pathways cannot expand, constraining future GP workforce distribution and sustainability.

Longstanding GP shortages have resulted in reduced access to timely care, increased emergency department presentations, and avoidable morbidity and mortality in rural and remote communities (3). Between 2023 and 2024, 46% people in these communities reported waiting more than 24-h for urgent medical care with a GP and this wait time was worse for people living with a long-term health condition (5). Despite substantial policy attention, the gap between supply and demand continues to widen. Driving this trend is an ageing GP workforce, increased population complexity due to ageing and chronic disease, and ongoing reliance on short-term international medical graduates and locums, which undermines continuity of care and local workforce sustainability (1, 3, 6).

Addressing maldistribution requires solutions that strengthen the training pipeline, particularly within rural settings. The Australian apprenticeship style GP training model depends fundamentally on supervision. Registrars, typically in their second and third postgraduate years, progress through supervised general practice placements to develop independent competence (7, 8). Evidence consistently demonstrates that high-quality supervision during early career stages improves clinical confidence, shapes professional identity, and importantly, increases the likelihood of rural practice after fellowship (9, 10).

However, while recruitment into rural training pathways has increased, supervision capacity has not kept pace. In rural and remote regions, approximately 30% of GPs provide supervision for more than 50% of registrars, meaning that the training capacity of entire regions can depend on a minority of clinicians. This concentration of supervisory responsibility creates vulnerability, if even a small number of supervisors retire or withdraw, local training opportunities can collapse (11, 12). Barriers that limit GP supervision include increased workload, limited financial viability, variable governance arrangements, and a lack of national oversight and recognition of the supervisory role (13, 14). These systemic bottlenecks constrain the number of registrars who can be trained rurally, ultimately restricting rural GP workforce growth.

Thus, GP supervisors represent a critical yet under examined determinant of rural workforce sustainability. A deeper focus on the enablers and barriers to supervision, particularly in high-need locations, is essential to inform future policy and strengthen the capacity of rural general practice to train and retain its workforce. This narrative review examines GP supervision as both an educational and workforce policy issue, synthesizing evidence from health professions education, workforce planning, and rural health literature to identify reforms required to support a sustainable supervisory pipeline. Much of the available literature examining GP supervision consists of cross-sectional surveys, workforce analyses, and qualitative studies. While these studies provide valuable insights into supervision experiences and workforce trends, causal relationships between supervisory interventions and workforce outcomes remain difficult to establish, highlighting the need for further longitudinal and intervention-based research.

## Main text

### The role of GP supervision

General Practice supervisors are central to addressing Australia’s rural GP workforce gap and ensuring its long-term sustainability. As the backbone of the apprenticeship-style general practice training model, supervisors are more than teachers, they are mentors who create an “educational alliance” that supports a registrar’s clinical skills, professional development, and personal growth (15). Through this relationship, supervisors influence not only competence but also professional identity, confidence, and career intentions.

Rural training exposure under skilled supervision is one of the strongest predictors of future rural practice (10, 16, 17). Rural immersion during medical education is associated with up to a four-fold increase in intention to pursue a rural career (16, 17). Importantly, it is supervisors who contextualize learning through real world rural practice: complex multimorbidity, broader scope of practice, and deep community connection (18). High quality supervision therefore not only enhances registrar competence but reinforces the appeal and viability of a rural professional identity.

The role of a GP supervisor is comprehensive and holistic. Clinical oversight ensures patient safety and progressive entrustment. Educational guidance enables registrars to translate theoretical knowledge into safe rural practice. At the personal level, supervisors provide mentorship that can support wellbeing, foster resilience, and encourage a commitment to lifelong learning (19–21). These responsibilities collectively shape registrars into competent, confident, and community-embedded practitioners.

Because the supervision model is capacity dependent, the future distribution of the GP workforce is directly linked to the availability of rural GP supervisors. In essence: **no** supervisors, no rural registrars, and no rural workforce pipeline. Strengthening supervision must therefore be recognized as a critical, strategic lever for addressing rural GP maldistribution.

### Issue at hand: comparison of supply, demand and projected shortfalls

Australia is entering a period of significant GP workforce contraction. The national GP workforce is ageing, with an average practitioner age of 50.6 years, and rural GPs being older again (1). Retirement intentions suggest that a substantial proportion of the current workforce will exit clinical practice within the next decade (5). This contraction is already visible. After a relatively stable period from 2019 to 2022, the number of practicing GPs declined sharply between 2022 and 2023, falling by 4,401 clinicians (a 14.2% reduction) (3). While partly reflecting the predictable effects of an ageing workforce, this post-pandemic drop also likely represents accelerated attrition associated with COVID-19–related burnout, workload intensification and shifts in career trajectories. The sudden loss of practicing GPs following the pandemic disproportionately affects rural areas, where a smaller and ageing supervisory cohort means that even modest workforce losses significantly reduce local training capacity.

Simultaneously, demand for GP services is increasing at a pace the system is not equipped to absorb. One in six Australians are now aged 65 years or older, and demographic projections indicate this proportion will continue to rise (3). The Parliamentary Budget Office forecasts health expenditure growth of $36 billion by 2028 - 2029, largely attributable to ageing and chronic disease burden (22). Rural communities, already characterized by geographic barriers, multimorbidity and limited specialist access, will be further disproportionately affected by this “double hit” of shrinking supply and escalating healthcare need.

To offset persistent maldistribution, rural and remote regions have become heavily reliant on international medical graduates (IMGs) and locum doctors, who comprise up to 40% of the GP workforce in some areas (23, 24). While essential for meeting immediate service demand, this dependence reflects a structural fragility. IMGs and short-term locums tend to higher turnover and reduced continuity, limiting the development of stable practice teams and creating challenges for community integration, quality improvement and registrar supervision (25–27).

Importantly, recent evidence indicates that IMGs are also substantially less likely to take on supervisory roles, even when they remain in rural practice. Catzikiris et al. found that IMG graduates had markedly lower odds of providing in-practice teaching or supervision (OR ≈ 0.36) compared with Australian-trained GPs, with lack of confidence identified as a key contributing factor (28). This suggests that increased reliance on IMGs does not translate into increased supervision capacity, further compounding the vulnerability of rural training pipelines.

Workforce sustainability ultimately depends on a strong rural training pipeline. However, supervision capacity is contracting at the same time that the need for supervisory roles is rising. As older GPs retire, the system simultaneously loses experienced clinicians and experienced supervisors, creating a dual bottleneck. Even if registrar recruitment increases, training cannot expand without sufficient supervisors in rural practices to provide clinical oversight, assessment and mentorship. Therefore, supervision is not an adjunct consideration but a central rate-limiting factor in addressing GP workforce shortfalls. Strengthening supervisory capacity must be recognized as a strategic priority for securing the future rural and remote workforce.

### Issue at hand: influence of high-quality supervision on workforce outcomes

The apprenticeship model of general practice training is effective only when supported by high-quality supervision. Supervisors shape registrars’ development through graded responsibility, feedback, and reflective learning, which together foster clinical confidence, professional identity formation and a commitment to lifelong learning (15, 17). These attributes are strongly linked to registrars remaining in general practice and feeling equipped to work independently in underserved settings.

Growing evidence demonstrates that supervision quality influences workforce distribution. Registrars who undertake rural training under experienced supervisors are more likely to practice rurally after fellowship (18). Exposure to the broader rural scope of practice, modeled by supervisors who are embedded in their communities, reinforces a sense of purpose, professional fulfillment and confidence in advanced skills (17).

Supervision standards embedded within fellowship pathways have measurable workforce consequences. Analysis of national workforce survey data shows that GPs with rural-focused fellowship training are significantly more likely to practice in rural and remote settings than non-vocationally trained GPs. This effect is strongest among ACRRM fellows, 56%–67% of whom practice rurally (OR 4.2, 95% CI 2.2–7.8), compared with more modest rural retention among RACGP fellows (25%–41%; OR 1.1, 95% CI 0.5–2.5) (4). This disparity likely reflects differences in training philosophy and rural embeddedness between fellowship pathways, including the requirement for supervisors to have substantial rural experience and to provide context-specific mentorship aligned with rural scope of practice (4, 20, 29). ACRRM’s longstanding training philosophy, including requirements for supervisors to have substantial rural experience and provide contextualized mentorship, is likely an important contributing factor to this effect (20, 29).

Taken together, these findings reinforce a simple but powerful conclusion. That is, when supervision is strong, registrars feel supported to stay, and rural training pathways produce rural doctors. Conversely, variability or insufficiency in supervision capacity undermines rural workforce outcomes regardless of training pathway investments. High quality supervision must therefore be considered a central mechanism through which rural workforce reform is achieved.

### Evidence-based reform: enablers of GP supervision

Strengthening supervision capacity requires building on the strong intrinsic motivations that many GPs already possess while addressing structural barriers that limit participation. Supervisors describe substantial personal and professional rewards from teaching, including enhanced clinical reasoning, continued skill development, and the satisfaction of contributing to the next generation of rural doctors (30, 31). Positive experiences of being supervised also create a self-reinforcing cycle, with registrars expressing a desire to emulate the mentors who shaped their careers and remain in rural practice themselves (32).

However, intrinsic motivation alone is insufficient to sustain supervision at the level required to address rural workforce shortages. Strategic incentives can amplify participation by ensuring that the time, effort and responsibility of supervisory roles are appropriately supported. Financial remuneration, protected teaching time, and accessible professional development programs have all been highlighted as high value enablers (6, 33). Recognition mechanisms, such as formal teaching titles, career advancement pathways, and accreditation standards, have the potential to also reinforce the status and identity of GP supervisors within the broader profession.

Workforce demographic shifts present both a challenge and an opportunity for supervision expansion. Female GPs now comprise over half of medical graduates and 43% of the practicing workforce yet they are less likely to take on supervision roles when workplace flexibility, childcare support, or professional confidence-building structures are lacking (4, 34). The supervisor identity issues identified by Catzikiris et al. including imposter syndrome, lack of confidence, and absence of clear pathways into supervisory roles, suggest that targeted support, mentoring, and structured supervisor development programs are essential to expanding participation across both IMG and female GP cohorts (28).

While increasing supervision capacity is essential, expansion must not come at the expense of supervision quality. Rapid increases in supervisory numbers without adequate preparation, training, or support may risk variability in educational experiences and compromise registrar development. The challenge is therefore not simply to recruit more supervisors but to develop systems that maintain educational quality while increasing capacity. Structured supervisor development programs, ongoing professional development requirements, mentorship for novice supervisors, and clearer national standards may help mitigate this risk.

Overall, the evidence indicates that the strongest approach combines intrinsic motivators with practical and financial supports, embedded within a system that recognizes supervision as a valued and professionally rewarding role. Enabling a broader cohort of GPs, particularly women, to assume supervisory responsibility represents one of the most impactful strategies for strengthening rural GP workforce sustainability.

### Challenges and barriers to GP supervision

1. Workforce capacity constraints and workload pressures

A major barrier to expanding supervision capacity is the limited number of rural GPs available to supervise. Rural GPs already work longer hours with greater on-call demands than their metropolitan counterparts, making it difficult to incorporate teaching into daily practice (32).

As the GP workforce ages and decentralized training expands, the number of registrars requiring supervision has increased supervisory demand in the context of a constrained and ageing rural workforce (6). Time pressures, increasing administrative tasks and high clinical demand reduce the feasibility of taking on supervisory responsibilities. Although government initiatives have sought to increase supervision participation, these efforts have often focused on numerical targets rather than the practical conditions required for sustainable supervision, reinforcing the need for enabling policies that reduce workload burden and support supervision as core clinical work rather than an additional obligation (6, 14, 18).

Addressing these constraints requires structural reforms that reduce the supervisory burden on individual clinicians. One approach is the partial off-loading of administrative and educational responsibilities to dedicated non-GP roles, such as practice-based clinical educators, who can support orientation, basic skills teaching, learning plan documentation, portfolio review and feedback collation, thereby freeing GP supervisors to focus on higher-level clinical teaching and oversight. Additionally, embedding funded, protected teaching time within weekly practice schedules would help alleviate the current pressure on supervisors to squeeze teaching into clinical gaps, improve educational consistency, and reduce the financial disincentives associated with reduced consulting time.

Professional and structural solutions are also needed to manage supervisory workload more equitably. A supervisor workload classification framework, based on registrar full time equivalent (FTE) load, would allow regulators and practices to identify high-burden supervisors and provide tailored financial or workforce supports, rather than relying on blanket incentives. Complementing this, a tiered supervision model, with primary and co-supervisors, similar to higher-degree research supervision structures would distribute workload across a broader supervisory team and create an accessible entry point for early-career GPs to begin developing supervisory skills.

Finally, policy levers must reflect geographic inequity and workload variation. A supervision incentive weighting scheme, aligned to MMM classifications and supervisory workload, would ensure proportionate remuneration for rural supervisors who have a high level of responsibility for trainees. Expansion of funded tele-supervision pathways would also provide essential relief to isolated practices, enabling high-quality supervision where onsite supervisory capacity is limited.

Together, these reforms would shift supervision from an individual burden carried by a small cohort of rural GPs to a more sustainable, system-supported function embedded across the rural primary care workforce.

2. Variability in supervision standards and oversight

Supervision quality is highly variable due to differences in local resources, practice structures and supervisory experience (14, 35). Although the RACGP and ACRRM provide guidance, neither establishes mandatory minimum standards for supervision or protected teaching time (12, 20, 21). Supervisors are largely responsible for defining supervisory structure within their practice, resulting in inconsistency in registrar experience and learning opportunities (31).

Registrars report generally high satisfaction with supervision but frequently note issues with inconsistent feedback and limited structured learning during busy periods (36). When supervisory arrangements rely too heavily on goodwill rather than systems of support, effective learning, registrar confidence and, critically, patient safety can be compromised (33).

Until recently, Australia lacked a nationally standardized and mandatory supervisor training framework. While registrar training requirements have long been governed through RACGP and ACRRM accreditation standards, formal preparation for the supervisory role itself was largely voluntary and locally determined. This contributed to variation in supervisory practices, confidence, and educational approaches across training sites.

3. Financial and structural

Business viability strongly influences whether a practice can supervise. Larger practices with multiple GPs more easily distribute supervisory responsibilities and accommodate additional teaching and administrative time (6). Smaller rural clinics often lack the physical space, support staff and financial margin to host registrars despite workforce need (37). Whilst hosting GP registrars may provide marginal financial benefit, this does not adequately offset increased workload, responsibility, and opportunity costs (14).

Current supervision standards within the Australian General Practice Training (AGPT) program generally limit a primary supervisor to overseeing no more than three GP registrars concurrently (21). While designed to safeguard patient safety and educational quality, this 1:3 supervisor-to-registrar ratio is applied consistently across practice settings and is not formally adjusted according to supervisor clinical full-time equivalent (FTE), practice size, rurality classification, or after-hours workload. Evidence indicates that supervision participation is strongly influenced by time pressure, administrative burden and practice viability (6, 14). In small rural practices, where workforce capacity and on-call demand are already high, uniform supervisory caps may therefore constrain training expansion and disproportionately affect practices operating with limited clinical redundancy (13, 31).

4. Lack of recognition and national coordination

General Practitioner supervisors frequently perceive their contribution as undervalued within the medical profession, particularly in comparison with hospital-based specialists (38). This can undermine motivation to take on or continue supervision roles. Additionally, fragmented governance between federal agencies and specialist colleges contributes to misalignment between training policy and workforce distribution goals (1). Poor visibility of rural supervisory opportunities and limited access to dedicated rural teaching support further constrain recruitment and retention of supervisors (35).

Collectively, these issues demonstrate that supervision cannot continue to rely on the goodwill and overstretch of a small cohort of rural clinicians. Instead, supervision must be redistributed, structurally supported, and financially recognized as a core workforce function. Implementing a coordinated package of reforms, combining protected teaching time, redistributed supervisory tasks, tiered supervision models, dedicated educator roles, and targeted financial incentives, would shift rural supervision from an individual burden to a sustainable, practice-embedded system. Strengthening supervision capacity in this way is essential for stabilizing the GP training pipeline and securing the long-term rural workforce.

Potential pilot initiatives could include funded protected teaching-time programs within MMM4-7 practices, regional co-supervision networks coordinated through Primary Health Networks and Regional Training Hubs, and targeted supervisor development pathways for women GPs and international medical graduates. Responsibility for implementation would likely require collaboration between the Australian Government Department of Health and Aged Care, RACGP, ACRRM, Regional Training Hubs, Rural Clinical Schools, and rural health services. Evaluation of these initiatives should include measures of supervisor retention, registrar satisfaction, training capacity, and subsequent rural workforce outcomes.

The recent formal recognition of Rural Generalism as a medical specialty represents a major step forward in strengthening rural medical training; however, its success will depend on the availability of experienced rural supervisors capable of supporting the broader scope and contextual demands of this training pathway. Without corresponding investment in supervision capacity, expansion of rural generalist training risks intensifying existing pressure on a small supervisory cohort rather than alleviating workforce shortages.

The evidence presented throughout this review suggests that strengthening rural GP supervision requires more than isolated workforce initiatives or financial incentives. Rather, supervision capacity is influenced by a combination of educational, organizational, professional, and policy factors that operate across multiple levels of the health system. Effective reform therefore requires a coordinated approach that supports supervisors, strengthens training infrastructure, and aligns workforce policy with educational capacity.

**FIGURE 1:**
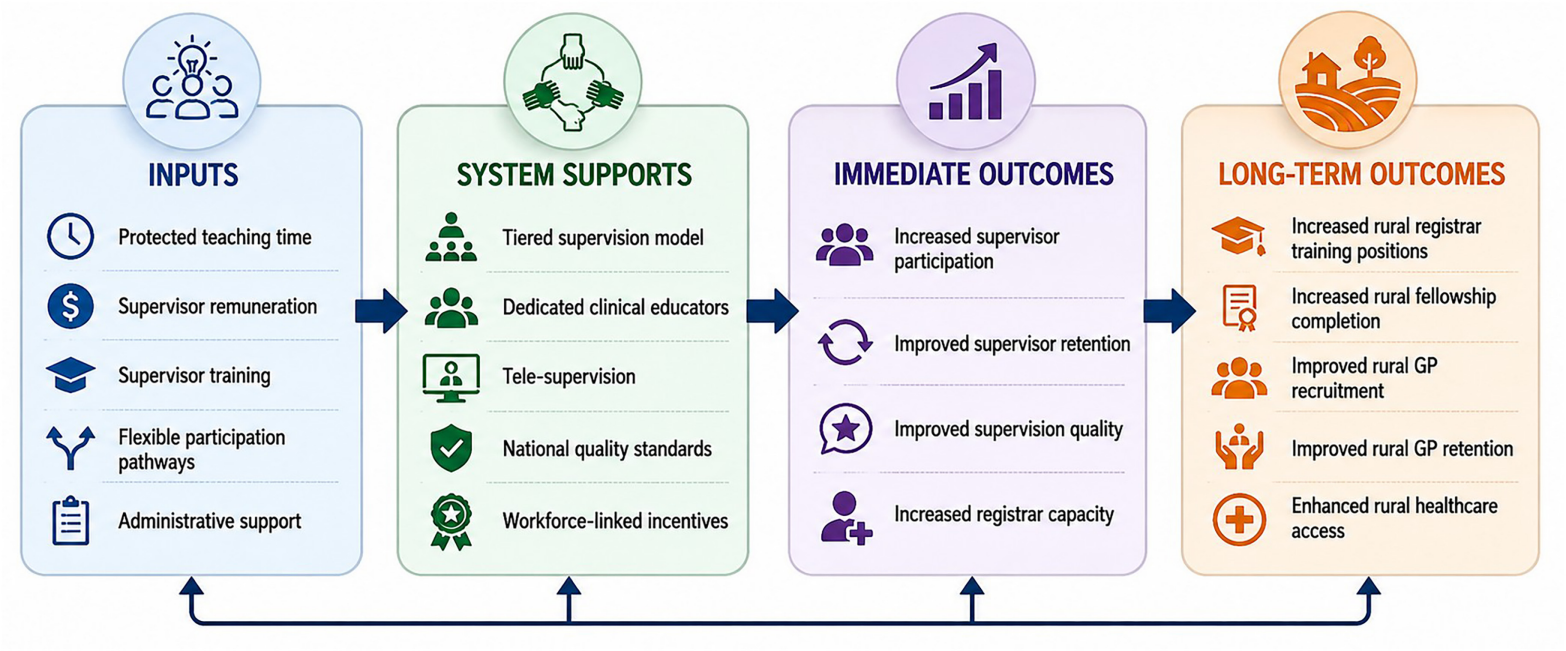
Proposed sustainable rural GP supervision framework: this illustrates how investments in supervisor support, training, remuneration, and infrastructure can create system-level enablers that improve supervision participation, retention, and quality. These improvements increase registrar training capacity and contribute to long-term workforce outcomes, including improved rural GP recruitment, retention, and healthcare access.

Figure 1 synthesizes the key findings of this review and presents a conceptual framework illustrating how targeted investments in supervision capacity may translate into improved rural workforce outcomes. The framework positions GP supervision as a critical intermediary mechanism linking workforce policy interventions with the long-term sustainability of the rural medical workforce.

This review has several limitations. As a narrative review, it does not employ the systematic search, selection, and quality appraisal processes associated with systematic reviews. Additionally, much of the available evidence examining GP supervision is observational in nature and originates from the Australian context, which may limit international generalisability. Nevertheless, the consistency of findings across workforce, educational, and policy literature strengthens confidence in the themes identified.

## Conclusion

As illustrated in Figure 1, general practice supervision is the structural backbone of Australia’s apprenticeship-style training model and a critical intermediary mechanism linking workforce investment with long-term rural workforce sustainability. This review has demonstrated that supervision quality and capacity directly influence registrar competence, confidence, and professional identity, all of which shape future workforce distribution. This review has demonstrated that supervision quality and capacity directly influence registrar competence, confidence, and professional identity, all of which shape future workforce distribution. The evidence consistently shows that registrars who train under experienced, well supported supervisors, particularly in rural contexts are significantly more likely to remain in those communities after fellowship.

Yet, supervision capacity in rural and remote areas is being eroded by systemic pressures: an ageing workforce, escalating workload demands, limited financial incentives, and the absence of coherent governance and recognition frameworks. These constraints collectively represent a critical bottleneck in the rural GP training pipeline. Without deliberate investment in supervision, expansion of rural training programs will continue to outstrip the system’s ability to sustain quality placements.

The current supervision paradigm is largely characterized by reliance on the goodwill and personal commitment of individual rural clinicians, with supervision often undertaken in addition to existing clinical workloads and with variable organizational support. This reactive model places responsibility for workforce development on a relatively small number of highly engaged practitioners and leaves training capacity vulnerable to retirement, burnout, and workforce attrition. In contrast, we propose a system-supported paradigm in which supervision is recognized as a core workforce function, supported through protected teaching time, structured career pathways, dedicated educational roles, and funding mechanisms aligned with workforce needs. Under this model, responsibility for training is embedded within the health system rather than dependent on individual effort alone.

Addressing this challenge requires a paradigm shift from short-term workforce fixes to long-term system building. Strategies that enhance supervisory capacity, such as protected teaching time, equitable remuneration, streamlined administrative processes, and recognition of supervisory expertise as a core professional competency, are essential. Policy frameworks must also accommodate the changing demographics of the GP workforce by supporting flexible and family friendly supervision models that engage the growing proportion of female practitioners.

Ultimately, strengthening GP supervision is not merely a training initiative; it is a strategic workforce intervention. By embedding supervision at the center of rural and remote GP training reform, Australia can create a self-sustaining cycle of mentorship, professional growth, and community continuity. Investment in supervisors today will determine the resilience, equity, and accessibility of rural healthcare for decades to come.
